# Role of Human Breast Cancer Related Protein versus P-Glycoprotein as an Efflux Transporter for Benzylpenicillin: Potential Importance at the Blood-Brain Barrier

**DOI:** 10.1371/journal.pone.0157576

**Published:** 2016-06-14

**Authors:** Yangfang Li, Qian Wu, Chen Li, Ling Liu, Kun Du, Jin Shen, Yuqin Wu, Xiaofen Zhao, Mei Zhao, Lingyun Bao, Jin Gao, Richard F. Keep, Jianming Xiang

**Affiliations:** 1 Department of Neonate, Kunming Children’s Hospital, Kunming, China; 2 Department of Neurosurgery, University of Michigan Medical School, Ann Arbor, United States of America; University of Cambridge, UNITED KINGDOM

## Abstract

While the blood-brain barrier (BBB) protects the brain by controlling the access of solutes and toxic substances to brain, it also limits drug entry to treat central nervous system disorders. Many drugs are substrates for ATP-binding cassette (ABC) transporters at the BBB that limit their entry into the brain. The role of those transporters in limiting the entry of the widely prescribed therapeutic, benzylpenicillin, has produced conflicting results. This study investigated the possible potential involvement of P-glycoprotein (P-gp) and breast cancer resistance protein (BCRP), two ABC transporters, in benzylpenicillin transport at BBB in human using MDCKII cells overexpressing those transporters as well as pharmacological inhibition. MDCKII cells overexpressing human BCRP (MDCKII-BCRP) but not those overexpressing human P-gp (MDCKII-MDR cells) had reduced [^3^H]benzylpenicillin uptake. Similarly, inhibiting BCRP increased [^3^H]benzylpenicillin uptake in MDCKII-BCRP cells, while inhibiting P-gp in MDCKII-MDR cells had no effect on uptake although there was evidence that benzylpenicillin is a substrate for canine P-gp. While inhibiting BCRP affected [^3^H]benzylpenicillin cell concentrations it did not affect transepithelial flux in MDCKII-BCRP cells. In summary, the results indicate that human BCRP and not human P-gp is involved in benzylpenicillin transport. However, targeting BCRP alone was not sufficient to alter transepithelial flux in MDCKII cells. Whether it would be sufficient to alter blood-to-brain flux at the human BBB remains to be investigated.

## Introduction

The blood-brain barrier (BBB) is mainly formed by the brain capillary endothelial cells and their linking tight junctions. It is a dynamic and complex interface between blood and the central nervous system (CNS) that regulates the exchange of many compounds between blood and brain, including xenobiotics[1, 2]. A critical component for BBB function is a group of ATP-binding cassette (ABC) efflux transporters that are highly expressed on the luminal, blood-facing, plasma membrane of brain capillary endothelial cells[3, 4]. These include P-gp (P-glycoprotein), MRPs (multidrug resistance proteins) and BCRP (breast cancer resistance protein). They function as ATP-driven efflux pumps for xenobiotics, including therapeutics, and endogenous metabolites.

P-gp has a very wide substrate spectrum mediating the export of a wide variety of drugs including antibiotics, anticancer agents, anti-HIV drugs, immunosuppressive drugs, antihistamines and analgesics[5, 6]. MRPs consist of 13 subfamily members (MPR1-MRP13) also transport a wide range of compounds including xenobiotics such as anticancer drugs, antibiotics and anti-HIV drugs[3, 7]. BCRP is widely distributed (brain, intestine, liver and kidney) and, like P-gp and MRPs, it transports a broad structurally and functionally diverse range of substrates including antibiotics, anticancer drugs and antivirals. There is a growing literature on the relative importance of BCRP at the human BBB[8, 9]. Although BCRP substrate specificity differs from P-gp and MRPs, there is substantial overlap[9–13]. While ABC transporters are neuroprotective when they deny entry of neurotoxic compounds into brain, they are also obstacles to CNS drug delivery. Changes in the transporter expression and transport activity can have a profound effect on pharmacotherapy. Therefore, they have increasingly become a target and a novel strategy in treating CNS diseases and brain protection[14–16].

Because of their broad spectrum and wide therapeutic index, beta-lactam antibiotics (e.g. benzylpenicillin, ampicillin, amoxicillin and ceftriaxone), are the most commonly used antibiotics in treating bacterial infections, including those of the CNS[17, 18]such as neonatal purulent meningitis, which has a high mortality rate and causes neurological sequelae and lifelong impairment[19, 20]. Beta-lactam antibiotic toxicity is less common, but can be severe and antibiotic resistance also often develops[21, 22]. Previous studies have indicated that benzylpenicillin barely crosses the BBB, but peripherally administration of high dose benzylpenicillin can cause seizures[22, 23]. It has been reported that some beta-lactam antibiotics, such as benzylpenicillin, ampicillin and ceftriaxone, are substrates of P-gp [24–27], which might account for low brain penetration. However, another study suggested that P-gp and BCRP are not involved in benzylpenicillin efflux transport in humans[28]. Therefore, the mechanisms regulating benzylpenicillin entry into brain are still unclear, particularly in relation to which ABC transporters may efflux benzylpenicillin at the human BBB.

The purpose of this study was to investigate the potential involvement of human P-gp and BCRP in benzylpenicillin transport by using MDCKII cell overexpressing human P-gp and BCRP as well pharmacological inhibition.

## Materials and Methods

MDCKII, MDCKII-MDR and MDCKII-BCRP cells were obtained from Dr. A. H. Schinkel (Netherlands Cancer Institute, Amsterdam, Netherlands). Cell culture medium and fetal bovine serum (FBS) was purchased from Thermo Fisher Scientific (Grand Island, NY, USA) unless otherwise specified. The P-gp inhibitor tariquidar was from Medkoo Biosciences, Inc. (Chapel Hill, NC, USA) and BCRP inhibitors, Ko143 and 197226, were from Sigma (St. Louis, MO, USA) and EMD Millipore (Temecula, CA, USA). [^3^H]Benzylpenicillin (25 Ci/mmol) and [^14^C]mannitol (55 mCi/mmol) were purchased from American Radiolabeled Chemicals, Inc. (St. Louis, MO, USA).

### Cell Culture

For [^3^H]benzylpenicillin uptake experiments, cells were cultured in 12-well plates as previously described[29]. Briefly, cells were grown in DMEM supplemented with 10% FBS in humidified incubator with 95% air-5% CO2 at 37°C. For [^3^H]benzylpenicillin transepithelial transport, MDCKII, MDCKII-MDR and MDCKII-BCRP cells were grown on 12-well transwell inserts[30, 31]. After ~36 hours, transepithelial electric resistance (TEER) was measured to determine the barrier’s tightness before use. Inserts with TEER values above 200 Ω.cm^2^ were selected to conduct the transport study.

### [^3^H]Benzylpenicillin uptake in plates

**Fig 1:**
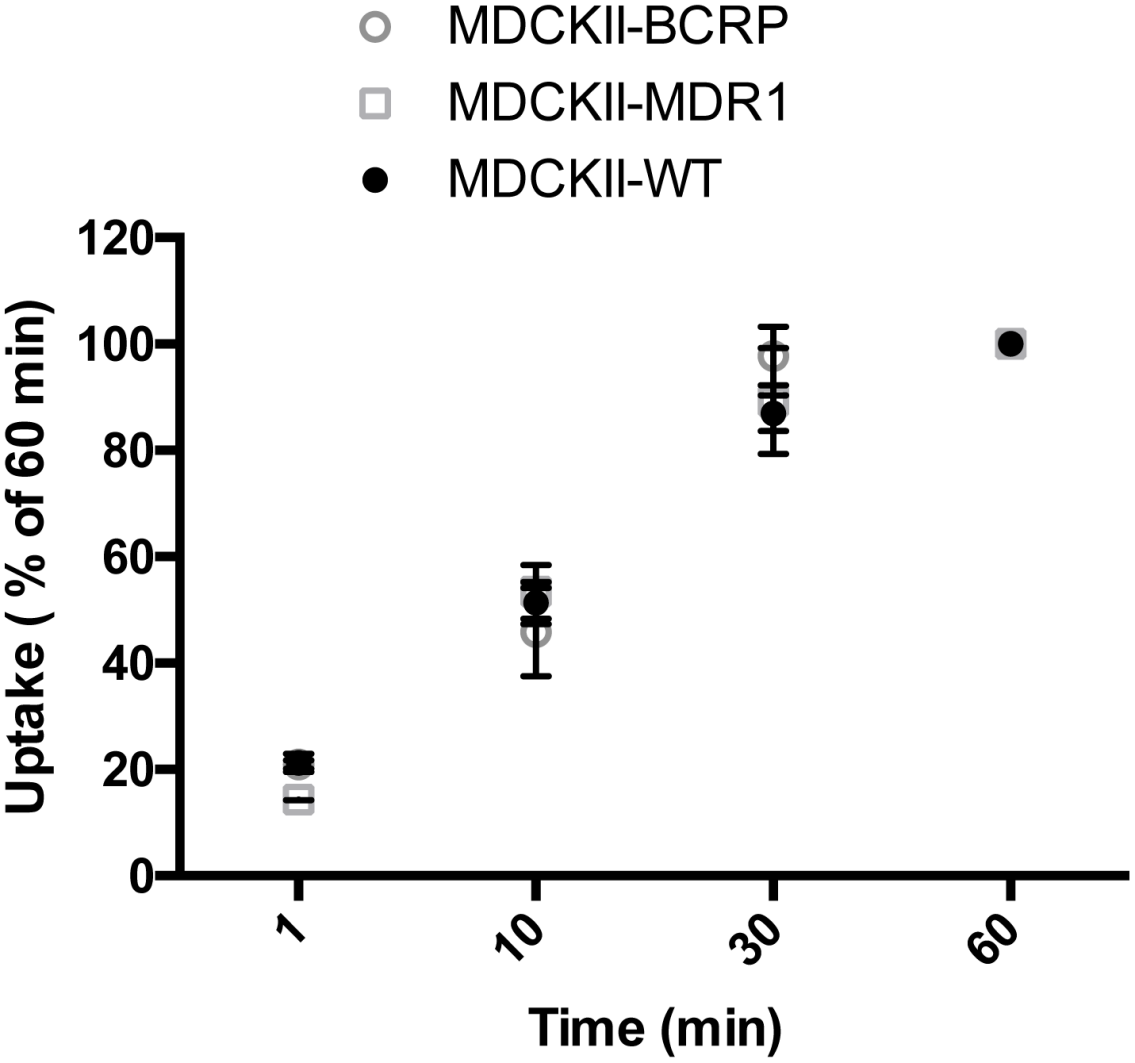
Time course of [^3^H]benzylpenicillin uptake in MDCKII, MDCKII-MDR and MDCKII-BCRP cells. The uptakes are expressed as a % of the 60 min value for each cell line. The uptake plateaued at ~60 min. Values are means +/- S.E., n = 3, each performed in triplicate.

[^3^H]Benzylpenicillin uptake was measured in MDCKII, MDCKII-MDR and MDCKII-BCRP cells. At the start of the experiment, the culture medium was removed and the cells were washed once with DMEM. Then, 1ml of uptake medium (DMEM) containing 0.1μCi [^3^H]benzylpenicillin and 0.05μCi [^14^C]mannitol (internal control), with or without inhibitors, was added to initiate uptake. After incubating at 37°C for 1 hour (chosen based on the time course experiments, Fig 1), the uptake medium was aspirated and the cells were rapidly washed with ice-cold PBS three times. Cells were solubilized with hyamine hydroxide and counted in a liquid scintillation counter (Beckman Coulter LS6500). Uptake was expressed per mg cell protein and [^14^C]mannitol was used to correct for extracellular contamination as described previously[32].

### [^3^H]Benzylpenicillin transepithelial transport in a transwell system

[^3^H]benzylpenicillin uptake was measured in MDCKII, MDCKII-MDR, MDCKII-BCRP cells as previously described [32]. At the beginning of the experiment, the culture medium was removed from the apical and basolateral chambers, and washed once with DMEM. Then, 0.5 ml of DMEM containing 0.1μCi [^3^H]benzylpenicillin and 0.05μCi [^14^C]mannitol, and transporter inhibitors (or without) was added to the apical chamber (apical to basolateral transport) or the basolateral chamber (basolateral to apical transport) while 0.5 ml of DMEM without radioisotope and inhibitor was added to the opposite chamber. Cells were then incubated at 37°C for 1 hour. To measure transepithelial transport, a 200μl of sample was taken from the opposite chamber and counted in a scintillation counter. To measure intracellular accumulation, the medium in both chambers was aspirated at the end of the experiment and the cells were rapidly washed three times in both chambers with ice-cold PBS. The filter plus cells was then placed in a scintillation vial and counted in a scintillation counter after being solubilized with hyamine hydroxide.

### Quantitative real time RT-PCR (qRT-PCR)

All of materials were from Thermo Fisher Scientific (Grand Island, NY, USA) unless otherwise stated. Total RNA was extracted from the cells using TRIzol Reagent according to the manufacture’s instructions. The concentrations and purity of RNA were estimated spectrophotometrically at 260 and 280 nm with NanoDrop 2000 (Thermo Fisher Scientific). cDNA was synthesized from 1μg of total RNA in a 20μL reaction mixture using a High Capacity cDNA Reverse Transcription Kit with RNase Inhibitor. The mixture was incubated at 25°C for 10 minutes, 37°C for 120 minutes, and 85°C for 5 minutes. Real-time PCR was carried out with Veriquest SYBR green master mix (Affymetrix, Inc, Cleveland, Ohio) in an Eppendorf Thermal Cycler. The reaction mixture was incubated at 95°C for 3 minutes and cycled 40 times from 95°C for 15 seconds to 60°C for 1 minute. The primers used for qRT-PCR are listed in Table 1.

**Table 1:** Primers used for qRT-PCR.

Protein name	Target gene name	GenBank Accession#	Forward primer 5’—3’	Reverse primer 5’—3’
P-gp, human	Abcb1	NM_000927.3	CCGAACACATTGGAAGGAA	CTTTGCCATCAAGCAGCAC
P-gp, canine	Abcb1	DQ068953.1	TTGCTGGTTTTGATGATGGA	CTGGACCCTGAATCTTTTGG
BCRP, human	Abcg2	AY017168	TTCGGCTTGCAACAACTATG	CTTTGCCATCAAGCAGCAC
BCRP, canine	Abcg2	DQ222459.1	GCTCTTCGGCTTCCAACAACTA	GTAACCCACTGACGCAAAGAACC
GAPDH,canine	GAPDH	AB038240.1	GCGGGGCCAAGAGGGTCATCAT	GCTTTCTCCAGGCGGCAGGTCAG

### Statistical analysis

Measurements were performed in triplicate and data from at least three independent experiments are presented as means ± SEM. Results were analyzed by t-test or one-way analysis of variance (ANOVA) followed by Dunnett *post hoc* test for comparisons to a single control or Tukey *post hoc* test for multiple comparisons between groups. A *P* value less than 0.05 was considered statistically significant (*P<0.05, **P<0.01 and ***P<0.001). All analyses were performed with Prism 5.0 (Graph Pad, San Diego, CA).

## Results

**Fig 2:**
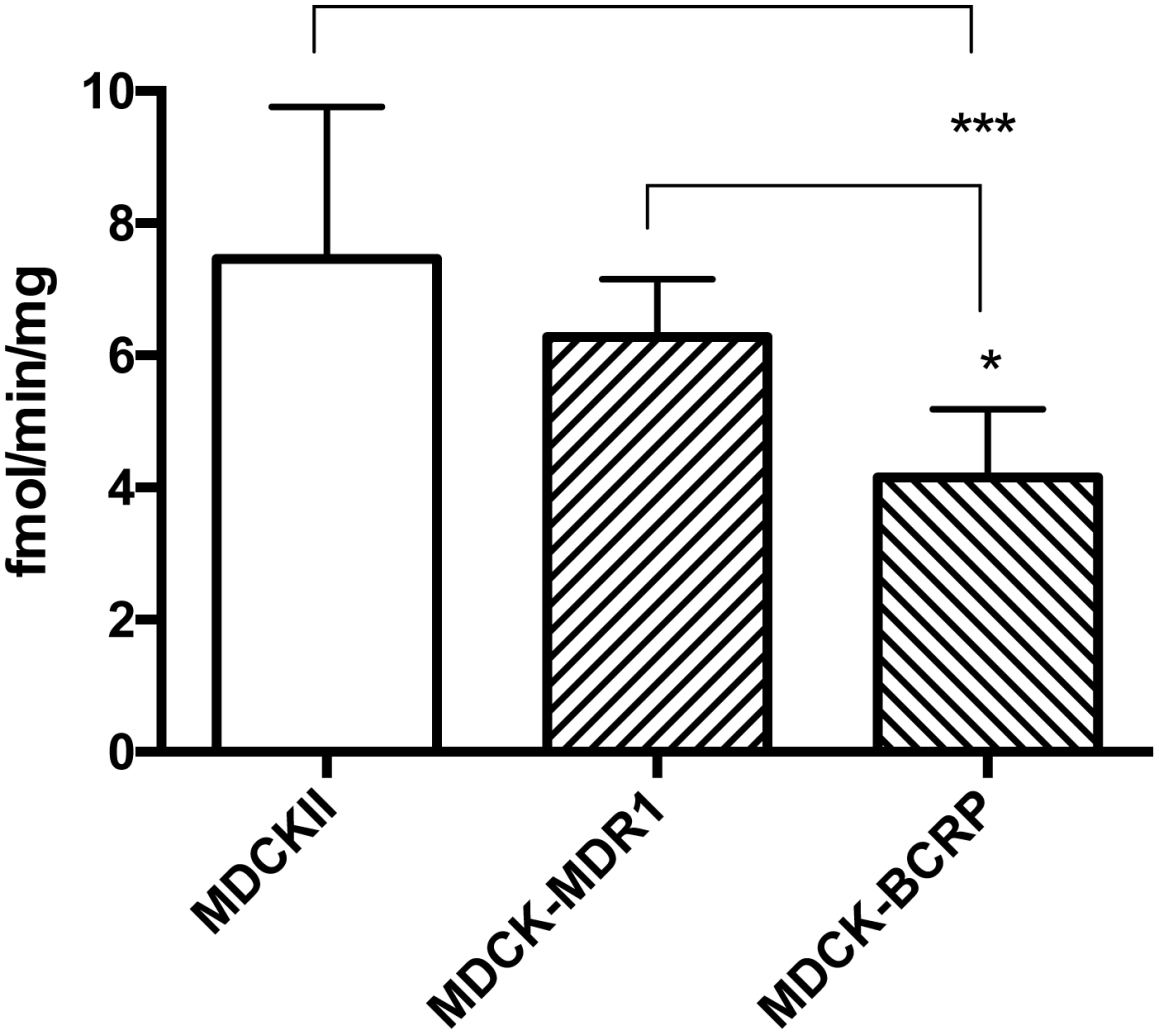
[^3^H] Benzylpenicillin uptake in MDCKII, MDCKII-MDR and MDCKII-BCRP cells. Values are means +/- S.E., n = 3, each performed in triplicate. * and *** indicate significant differences from comparison between different cells at the P<0.05 and P<0.001 levels, respectively.

The cellular uptake of [^3^H]benzylpenicillin was examined in WT-MDCKII (wildtype canine epithelial cell line), MDCKII-MDR (that cell line with human P-gp (MDR) overexpression) and MDCKII-BCRP (that cell line with human BCRP overexpression) cells in the absence and presence of P-gp and BCRP inhibitors. In the absence of inhibitors, overexpression of human P-gp (MDCKII-MDR) did not decrease the uptake of [^3^H]benzylpenicillin compared to WT cells (Fig 2) as would have been expected if benzylpenicillin was a human P-gp substrate. In contrast, overexpression of human BCRP (MDCKII-BCRP) did result in significantly reduced [^3^H]benzylpenicillin uptake (Fig 2), suggesting that benzylpenicillin is a BCRP substrate.

**Fig 3:**
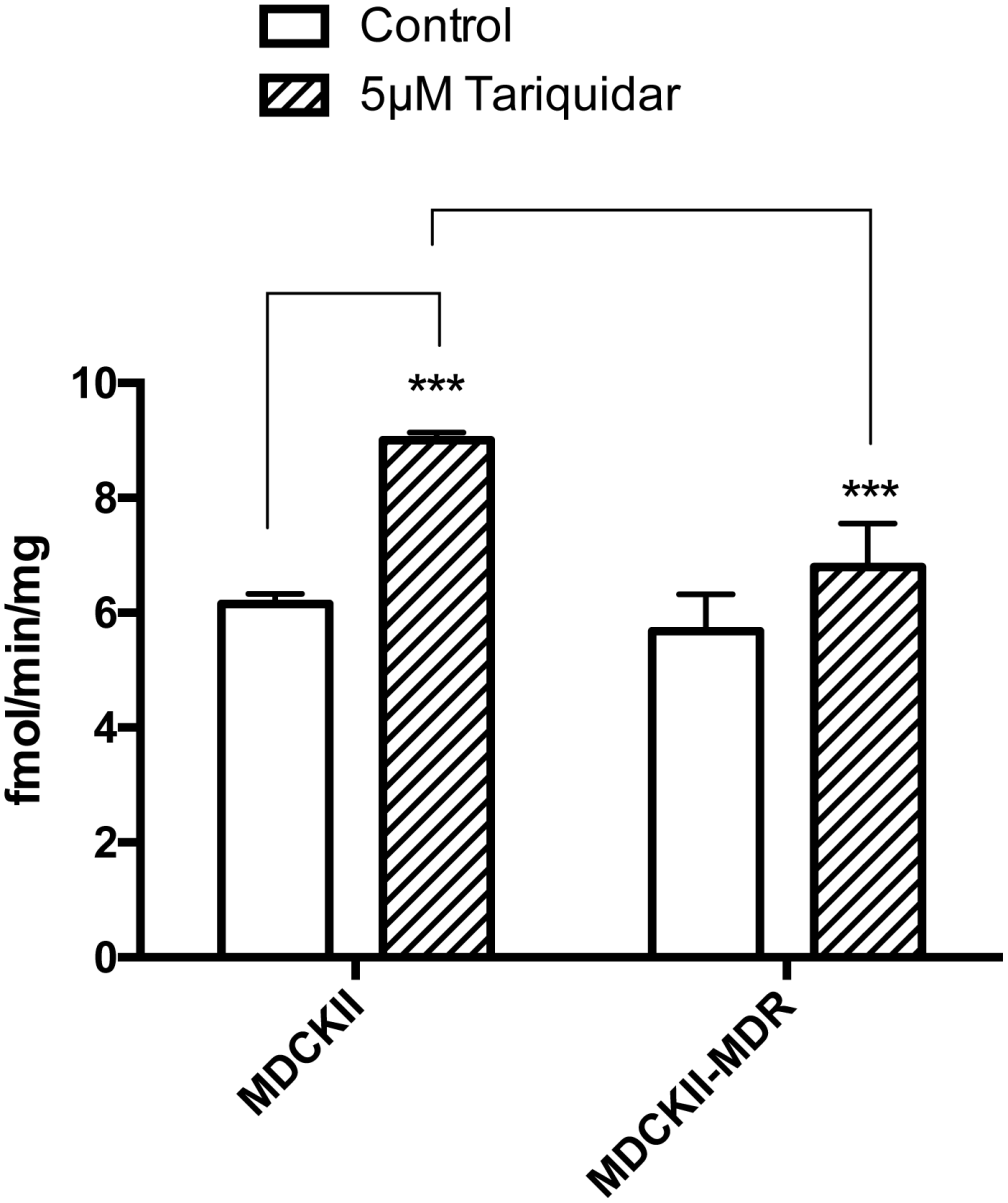
[^3^H] Benzylpenicillin uptake in MDCKII and MDCKII-MDR cells. Values are means +/- S.E., n = 3. *** indicates significant differences from comparison between two different cells and groups at the P<0.001 levels.

In WT-MDCKII cells, tariquidar (5μM), a P-gp inhibitor, increased [^3^H]benzylpenicillin uptake by1.46 ± 0.04 fold. In contrast, in MDCKII-MDR cells, benzylpenicillin uptake was not significantly changed. This suggests that that benzylpenicillin is a substrate of canine but not human P-gp (Fig 3). It should be noted that expression of human P-gp in MDCKII cells greatly suppresses the expression of endogenous canine P-gp[33].

**Fig 4:**
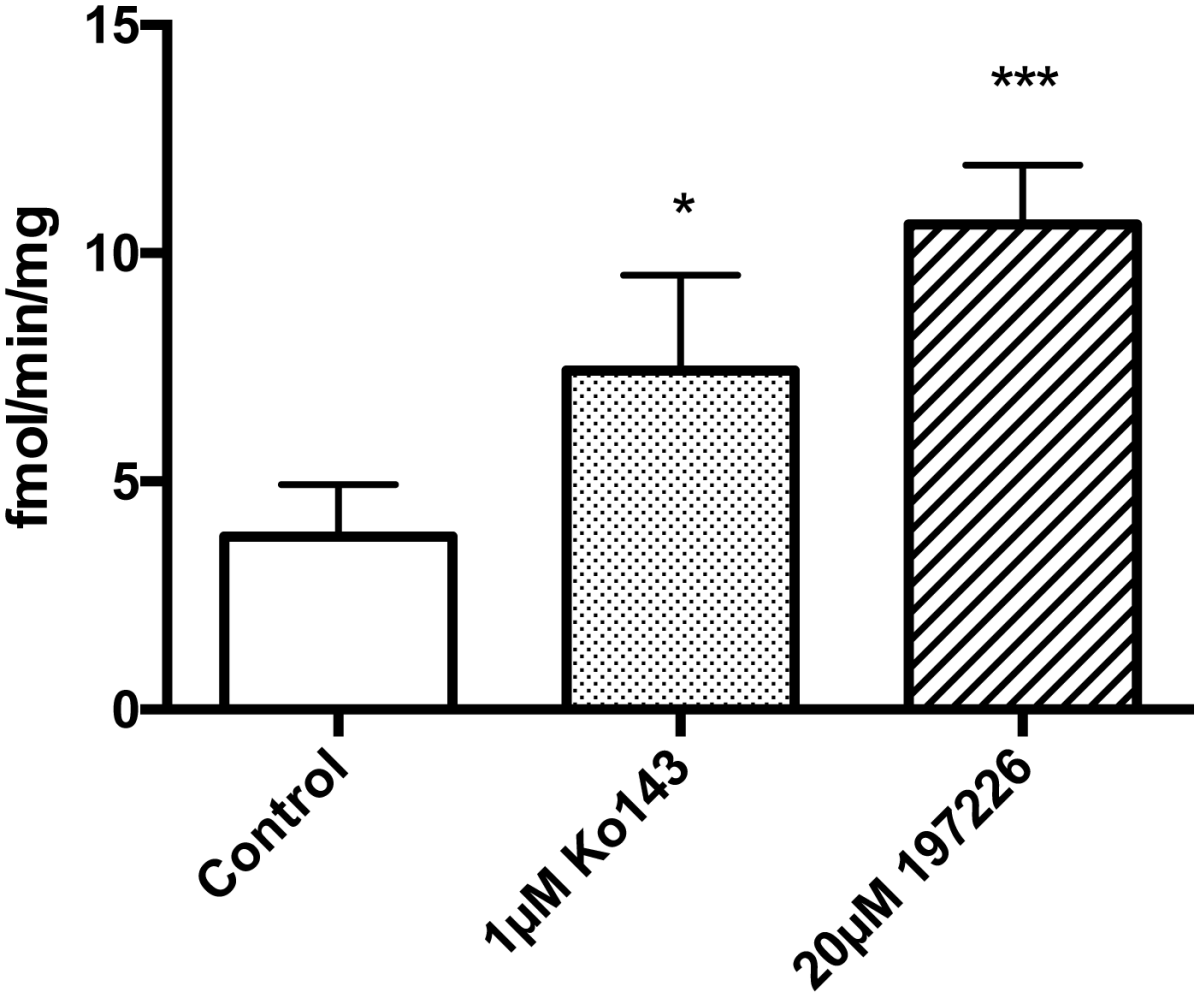
[^3^H] Benzylpenicillin transepithelial uptake in MDCKII-BCRP cells. Values are means +/- S.E., n = 3. * and *** indicate significant differences from control at the P<0.05 and P<0.001 levels, respectively.

Two BCRP inhibitors, Ko143 (1μM) or 197226 (20μM), had no effect on the uptake of [^3^H]benzylpenicillin into wild type MDCKII cells (data not shown). In contrast, in MDCKII-BCRP cells, the two inhibitors increased [^3^H]benzylpenicillin uptake (Ko143 by 1.6 ± 0.2 fold and 197226 by 3.8 ± 0.3 fold; Fig 4). These results indicate that human BCRP can efflux [^3^H]benzylpenicillin.

**Fig 5:**
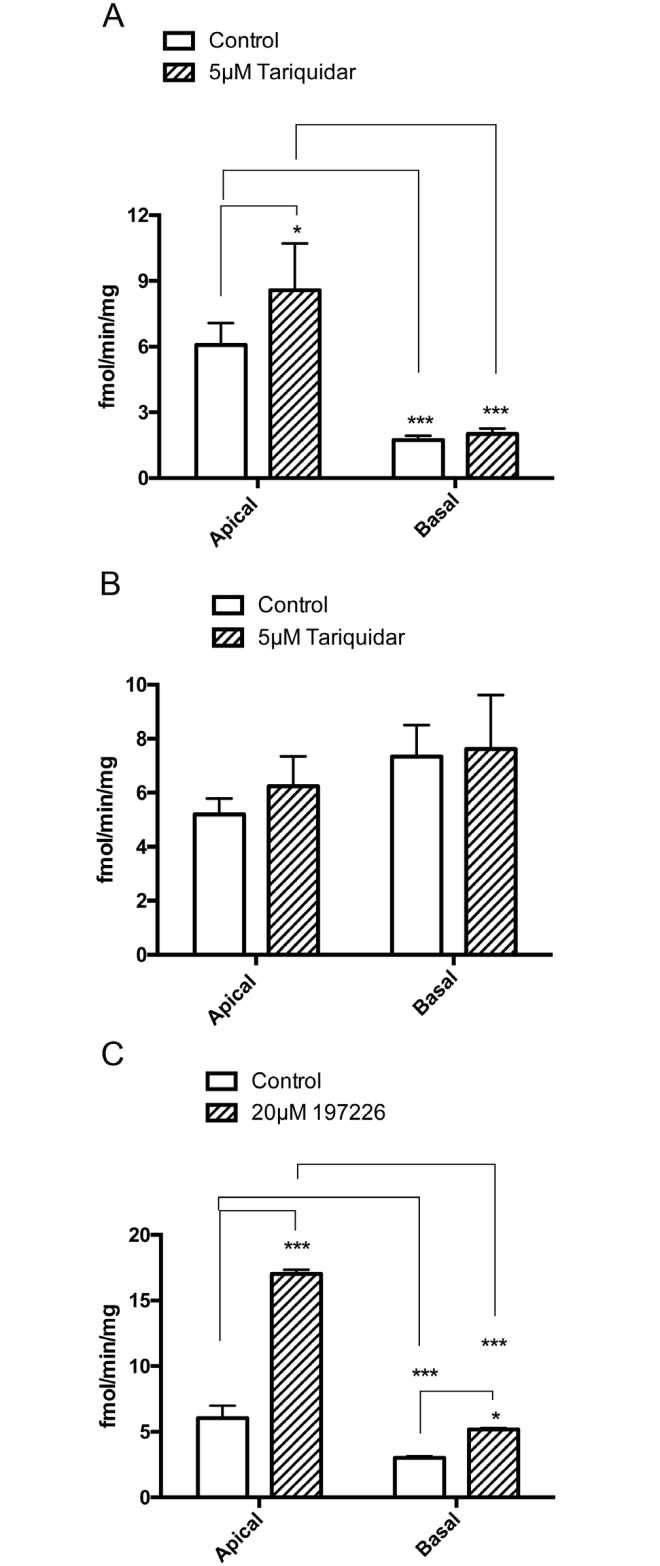
[^3^H] Benzylpenicillin uptake in transwell in MDCKII (A), MDCKII-MDR (B) and MDCKII-BCRP (C) cells. Values are means +/- S.E. n = 3. * and *** indicate significant differences from comparison between different groups at the P<0.05 and P<0.001 levels, respectively.

The transepithelial transport of [^3^H]benzylpenicillin was measured in a Transwell system in the apical to basolateral (A to B) and basolateral to apical (B to A) directions, with cellular accumulation determined at the end of the experiment. In WT MDCKII cells, the cellular accumulation of [^3^H]benzylpenicillin was markedly greater across the apical membrane than the basal membrane (Fig 5A). The apical, but not the basal, uptake was enhanced by tariquidar. In MDCKII-MDR cells neither apical nor basal uptake was affected by tariquidar (Fig 5B). The effects of tariquidar on apical [^3^H]benzylpenicillin uptake in WT MDCKII and MDCKII-MDR cells grown on transwells were similar to that found on plated cells. As with the WT MDCKII cells, the cellular accumulation of [^3^H]benzylpenicillin was markedly greater across the apical membrane compared to the basal membrane in MDCKII-BCRP cells (Fig 5C). Treatment with the BCRP inhibitor 197226 greatly enhanced the apical uptake of [^3^H]benzylpenicillin again supporting the findings from plated cells that apical BCRP acts as an efflux transporter for benzylpenicillin.

**Fig 6:**
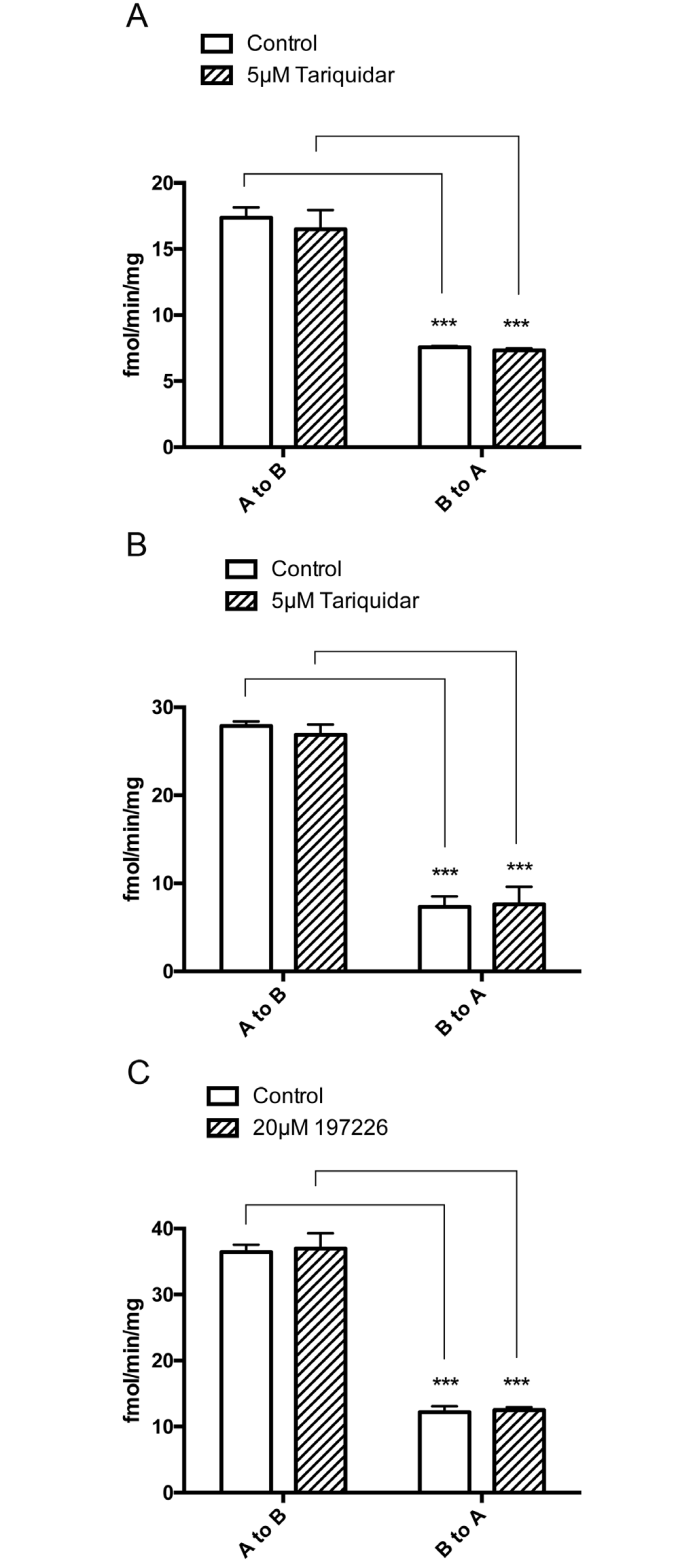
[^3^H] Benzylpenicillin transepithelial flux in MDCKII (A), MDCKII-MDR (B) and MDCKII-BCRP (C) cells. Values are means +/- S.E. n = 3. *** indicates significant differences from comparison between different groups at the P<0.05 and P<0.001 levels, respectively.

In all three cell types, the A to B transepithelial transport of [^3^H]benzylpenicillin exceeded the B to A transport (Fig 6). The P-gp inhibitor, tariquidar, did not affect transepithelial transport in either direction in WT-MDCKII or MDCKII-MDR cells (Fig 6A & 6B). The presence of the BCRP inhibitor, 197226, also did not affect transepithelial transport in either direction in MDCKII-BCRP cells despite it markedly increasing cellular [^3^H]benzylpenicillin uptake (Fig 6C).

To make sure that the MDCKII cells overexpressing MDR1 and BCRP had functionally active transporters, the uptake of known substrates was measured in those cell lines in the presence and absence of transporter inhibitors. In the MDCKII-MDR1 cells, the uptake of Rhodamine 123, a fluorescent MDR substrate, was increased 11.6+/-0.4 fold in the presence of the MDR inhibitor tariquidar (5 μM). Similarly, the uptake of [3H]vinblastine, another MDR substrate, was increased 24.5+/-2.5 fold by tariquidar. These results indicate that MDR is active in this cell line serving to keep the level of substrates low in the cells. For MDCKII-BCRP cells, the uptake of Hoechst 33342, a BCRP substrate, was examined in the presence or absence of the BCRP inhibitor Ko143 (1 μM). Ko143 increased Hoechst 33342 uptake 3.8+/-0.4 fold again indicating that BCRP is active in this cell line serving to keep the level of substrates low in the cells. The lesser fold effect of Ko143 on Hoechst 33342 uptake compared to the effect of tariquidar on the MDR substrates, may reflect the relatively lower expression of human BCRP in the MDCKII cells compared to MDR1 (Table 2). It should be noted that Ko143 had no effect on Hoechst 33342 uptake in MDCKII-WT cells. That is in agreement with the absence of canine BCRP in those cells (Table 2).

**Table 2:** mRNA expression of canine and human P-pg and BCRP (ΔΔCT) in the three MDCK cell lines.

	MDCKII-WT	MDCKII-MDR1	MDCKII-BCRP
Canine Pgp	0.55±0.15	0.27±0.04	0.49±0.15
Human Pgp	0	38.7±4.9	0
Canine BCRP	0	0	0
Human BCRP	0	0	4.9±1.09

To exclude possible involvement of endogenous transporters in benzylpenicllin transport in the cells and to confirm that BCRP is the main transporter mediating benzylpenicillin transport, the expression of canine/human P-gp and BCRP was examined in the three MDCK cell lines by qRT-PCR. As shown in Table 2, no mRNA of canine BCRP was found in all of the cell lines. The expression of canine P-gp mRNA in MDCKII and MDCKII-BCRP cells was almost exactly same while the expression of canine P-gp in MDCKII-MDR1 cells was about 50% less than the wild type cells because of the overexpress of human P-gp. The latter confirms prior results of Kuteykin-Teplyakov et al. [33]. In the MDCKII-BCRP cells, the expression of human BCRP mRNA was 10 times higher than the expression of canine P-gp mRNA.

## Discussion

The current study indicates that benzylpenicillin is a substrate of human BCRP. In contrast, it is not a substrate for human P-gp, although apparently it is for canine P-gp. The data indicate that targeting BCRP alone is not enough to affect transepithelial benzylpenicillin flux in MDCKII-BCRP cells. Whether it would be sufficient to alter blood-to-brain flux at the human BBB remains to be investigated.

Benzylpenicillin, one of the beta-lactam antibiotics, has been used to treat bacterial infections, including CNS infections such as neonatal purulent meningitis. Benzylpenicillin has a relatively low molecular weight of 372 and many reports have suggested it enters brain[22]. However, Rousselle et al. have shown that benzylpenicillin can barely cross BBB and this can be dramatically changed after coupling with SynB1 vector[23]. We examined the potential role of P-gp and BCRP in that low brain distribution. P-gp and BCRP are two major ABC efflux transporters abundantly expressed at the apical membrane of brain capillary endothelial cells. They act to limit the entry of a range of xenobiotic into brain[8, 9]. Both of them have a wide variety of substrates including many therapeutic drugs, such as antibiotics and anticancer agents. In terms of ABC transporter involvement in benzylpenicillin efflux transport, data from previous studies are controversial and contradictory. Poelarends et al. reported that benzylpenicillin and ampicillin are P-gp substrates after examining the effect of LmrA expression on the relative antibiotic resistance of E. Coli CS1562[25]. Bacterial LmrA is a structural and functional homolog of human P-gp. However, results from the current study showed that inhibiting P-gp with tariquidar, did not increase [^3^H]benzylpenicillin entry into MDCKII-MDR cells, indicating that human P-gp is not involved in benzylpenicillin efflux. There was an increase in benzylpenicillin uptake into wild-type canine MDCKII cells in the presence of tariquidar, which suggests that benzylpenicillin is a substrate for canine P-gp. Differences in substrate affinity between rodent and human P-gp have been noted previously[34–36]. One of the effects of overexpressing human P-gp in MDCKII cells is a marked suppression of endogenous canine P-gp ([33] and Table 2), which may explain why tariquidar had no effect on [^3^H]benzylpenicillin in MDCKII-MDR cells.

Previous studies by Choi et al. have shown that benzylpenicillin is not involved in BCRP-mediated mitoxantrone (a substrate of BCRP) efflux in competition experiments, indirectly suggesting that benzylpenicillin is not a substrate of BCRP[28]. However, our data showed that benzylpenicillin uptake into MDCKII-BCRP cells was decreased compared to WT cells and that it was significantly increased by 197226, a selective inhibitor of BCRP, specifically in MDCKII-BCRP cells. These results indicate that benzylpenicillin is a BCRP substrate.

Canine MDCKII cells expressing human transporters and grown on transwells have been used extensively to study the potential impact of transporters on drug disposition[37]. For example, an increase in the ratio of drug transport from B to A/A to B in MDCKII-MDR cells compared to WT MDCKII cells, or the presence of a transport inhibitor, is indicative of a P-gp substrate. In the current study, tariquidar did not affect transepithelial flux of [^3^H]benzylpenicillin in WT MDCKII or MDCKII-MDR cells. Interestingly, the BCRP inhibitor 197226 did not alter transepithelial flux in either the B to A or the A to B direction even though it increased cellular accumulation. This may reflect which is the rate limiting step for transepithelial transport across the cells; i.e. for B to A transport this may be basal uptake rather than the apical efflux.

It should be noted that there are dangers in extrapolating these transepithelial flux data to the BBB in vivo as the impact of BCRP inhibition on benzylpenicillin entry into brain will depend on the array of transporters for benzylpenicillin in the two systems and their affinity for benzylpenicillin. For example, benzylpenicillin is a substrate of rat organic anion transporter 3 (OAT3) that is involved in eliminating benzylpenicillin at the BBB[38]. In vivo data on BBB transport in BCRP KO mice might be informative, but our findings suggesting there may be species differences in P-gp benzylpenicillin transport would complicate interpretation. Definitive data would only be obtainable in human patients.

In summary, we found in this study that human BCRP rather than P-gp is involved in benzylpenicillin transport. Targeting that transporter may be a way of increasing benzylpenicillin entry into brain to treat infections although our transepithelial data suggest that it may be necessary to target more than one transporter.
